# Correlation of chronological, skeletal and dental (Demirjian method) ages in an Iranian growing orthodontic population – a cross-sectional study

**DOI:** 10.1038/s41598-026-39319-4

**Published:** 2026-02-09

**Authors:** Mehraneh Mohammadian-Rastani, Fatemeh Gorjizadeh, Seyedeh Roghayeh Panahi, Ali Mousavizadeh

**Affiliations:** 1 Dentistry Student, School of Dentistry, Yasuj University of Medical Sciences, Yasuj, Iran; 2 Department of Orthodontics, School of Dentistry, Yasuj University of Medical Sciences, Yasuj, Iran; 3 Department of Oral and Maxillofacial Radiology, School of Dentistry, Yasuj University of Medical Sciences, Yasuj, Iran; 4 Department of Biostatistics and Epidemiology, School of Health, Social Determinants of Health Research Center, Yasuj University of Medical Sciences, Yasuj, Iran

**Keywords:** Chronological age, Dental maturity, Skeletal maturity, CVMS, Demirjian method, Growth modification treatment, Anatomy, Diseases, Health care, Medical research

## Abstract

Growth modification treatments are a type of orthodontic treatment used for patients who are still growing. The treatment plan and timing for these interventions are generally based on the chronological age of patients; however, achieving the most desirable outcome requires a thorough understanding of the physical development of children and adolescents. The aim of this study is to examine the correlation between chronological age and physical development in children and adolescents requiring orthodontic treatment. To assess physical development, both dental age and skeletal age were evaluated. Panoramic and lateral cephalometric radiographs of 194 orthodontic patients aged 7 to 17 years were randomly selected from a dental clinic in Gachsaran. Dental maturity was assessed using the Demirjian method, while skeletal maturity was evaluated using the cervical vertebral maturation stage (CVMS) method described by Baccetti. Data analysis was conducted using SPSS software version 27. The correlation between variables was calculated using Pearson’s correlation coefficient and Spearman’s rank correlation. The mean dental age of patients was found to be higher than their chronological age. Notably, the strongest correlation between dental age and chronological age for both males and females were observed at the first cervical vertebral maturation stage (CVMS1) highlighting its potential clinical relevance for early growth assessment. The positive Pearson correlation between dental age and chronological age was 0.894 in males and 0.876 in females. Moreover, a strong and significant positive correlation was found between dental age and skeletal age, with a Spearman’s rank correlation coefficient of 0.779 in males and 0.785 in females. Additionally, a strong and significant positive correlation was observed between chronological age and skeletal age, with a Spearman’s rank correlation coefficient of 0.783 in males and 0.707 in females. The results of this study indicate a significant positive correlation between chronological age and both methods of assessing physical development (dental age and skeletal age). The Demirjian method appears to overestimate dental age in the study population, suggesting a need to reassess its validity in Iranian population. Furthermore, the strongest correlation between chronological age and dental age was observed at the CVMS1 for both genders. These insights should be taken into account when planning growth modification treatments.

## Introduction

Growth modification treatment is indicated in growing orthodontic patients and aims to correct skeletal disharmonies by utilizing the patient’s remaining growth potential^1^. Since the success of growth modification is highly dependent on treatment timing, accurate assessment of skeletal maturity is essential to identify the optimal period for intervention. The biological basis of growth modification is closely related to physical maturity, particularly the timing of the pubertal growth spurt. However, chronological age alone is an unreliable indicator of physical maturity^2^. As a result, developmental age has been introduced as an alternative measure, focusing on the maturity of specific body tissues. Several methods are available for assessing developmental age, with two common approaches in dentistry being dental age and skeletal age^3^.

Both dental and skeletal development are regulated by complex interactions between genetic determinants and environmental influences, including socioeconomic status, body mass index, and endocrine function. Notably, skeletal maturation appears to be more susceptible to environmental and systemic factors, whereas dental development tends to follow a more genetically predetermined pattern^4–6^.

Among biological markers used for age estimation, dental structures are considered particularly reliable due to their regular and well-defined formation stages, as well as their relative resistance to external environmental and physiological variations. Compared with other maturity indicators, teeth demonstrate lower interindividual variability, contributing to greater consistency and accuracy in forensic and anthropological evaluations^7,8^. Several techniques estimate dental age based on tooth mineralization visible in panoramic radiographs. Among these, the Demirjian, Willems, and London Atlas methods are the most widely used^9^.

Skeletal age is commonly assessed using either the Hand-Wrist method or Cervical Vertebral Maturation (CVM)^10^. Since lateral cephalometric radiographs are routinely taken in orthodontic practice, the CVM method is often preferred to minimize unnecessary radiation exposure^11^. Determining skeletal age is especially important when planning treatment for patients with different malocclusions. For example, Class II patients achieve the best outcomes when treated during the CVMS3 stage, while treatment for Class III patients with maxillary retrusion is most effective during CVMS1 or CVMS2^12^.

Also research shows that children today are reaching puberty earlier than previous generations. This trend is associated with multiple factors, including socioeconomic disadvantage, which correlates with accelerated biological aging, and childhood obesity, which is linked to increase linear growth and earlier puberty^13,14^. Several studies have emphasized that chronological age alone does not accurately reflect an individual’s biological maturation, prompting the use of dental and skeletal indicators as complementary assessment tools^7,15^. Consequently, numerous investigations have explored the relationships among these biological age markers.Previous studies have reported varying degrees of correlation among chronological age, dental age, and skeletal maturity, with results strongly influenced by ethnic, genetic, and environmental factors^16^.

These variations highlight the need for population-specific data to guide growth modification treatments. Because few studies have simultaneously evaluated chronological age, dental age, and skeletal maturity in Iranian adolescents, this study aimed to assess the correlations among these measures in orthodontic patients to support more effective clinical decision-making.

## Materials and methods

### Sample collection

This cross-sectional study was conducted at an orthodontic clinic in Gachsaran. Patient records of individuals aged 7–17 years were screened. The upper age limit of 17 years was selected based on the original Demirjian method and subsequent validation studies^17^. Eligible records were required to include both panoramic and lateral cephalometric radiographs, taken within one week of each other and of high diagnostic quality. For the purpose of chronological age calculation, the date of the panoramic radiograph was considered the reference point. Since all radiographs were obtained prior to the initiation of orthodontic treatment, variations in treatment type did not influence skeletal or dental assessments. Records were excluded if permanent teeth were missing on the left side of the mandible (excluding third molars). Patients with a history of head or neck trauma, systemic disease, or medication use affecting normal growth were also excluded. Replacement candidates were selected as needed. The required sample size for the correlation analysis was calculated using Fisher’s Z-transformation for correlation coefficients. Based on an expected correlation derived from a previous study^18^, the total sample size was determined as *N* = 194 to achieve a significance level of 0.05 and 80% power, ensuring adequate statistical reliability.

No new radiographs were obtained for the purposes of this research; only existing files meeting the inclusion criteria were used, thereby avoiding unnecessary radiation exposure in children.

### Assessment of dental and skeletal maturity

Dental maturity was assessed using Demirjian’s method, which estimates dental age based on the mineralization and root development of teeth rather than eruption sequence^17^. Each patient’s panoramic radiograph was examined for the development of seven left mandibular teeth (incisors, canine, premolars, and molars, excluding third molars). Each tooth was assigned a developmental stage from A to H as illustrated in the original publication, where Stage A indicates the least and Stage H the most advanced mineralization. This approach relies on morphological changes occurring throughout the stages of dental development, including crown formation, initial root development, root length, and the status of apical closure. Each stage, considering the type of tooth and the patient’s sex, corresponds to a specific numerical score according to the reference tables. The total score of the seven teeth was then calculated, and the corresponding dental age, which is a quantitative variable, was determined using the sex-specific tables provided in the original study^17^.

Skeletal maturity was evaluated using the Cervical Vertebral Maturation (CVM) method described by Baccetti^19^. This method assesses the morphology of the second, third, and fourth cervical vertebrae on lateral cephalometric radiographs. In this method stage 1 represents the least mature vertebrae and stage 6 the most advanced. CVM stages are defined by morphological changes in the lower borders and shapes of C2–C4 vertebrae, ranging from flat, trapezoidal vertebrae indicating a growth peak more than two years away (CVM1) to vertically rectangular vertebrae indicating completion of the growth peak more than two years earlier (CVM6)^20^.

All radiographs were evaluated by a single trained examiner using a negatoscope under natural light. To ensure consistency, all images were obtained from the same radiology center with identical settings. Approximately 15 radiographs were assessed per session to minimize fatigue. To ensure examiner calibration and reliability, all images were re-evaluated by the same examiner after a two-week interval, blinded to the initial assessments. Any discrepancies were resolved by a second independent, board-certified orthodontist. Intra-examiner reliability was excellent, with a weighted kappa of 0.87, indicating highly consistent measurements.

### Statistical analysis

The study included 194 orthodontic patients (149 females, 45 males). Skeletal age (CVM stage) was treated as an ordinal variable, while dental and chronological ages were quantitative. Pearson’s correlation coefficient was used for relationships between quantitative variables, and Spearman’s rank correlation was applied for associations involving the ordinal skeletal age. All analyses were performed using SPSS version 27.

Ethical approval 1-All methods were carried out in accordance with relevant guidelines and regulations.

2-The experimental protocols were approved by the Institutional Ethics Committee of Yasuj University of Medical Sciences, approval number IR.YUMS.REC.1403.006.

3-Due to the use of existing radiographs and patient records, the requirement for informed consent was waived by the ethics committee.

## Results

### Average chronological age and dental age

The study sample consisted of adolescents (males and females) from Gachsaran with a mean chronological age of 13.7 years (± 2.08). Using the Demirjian method, the dental age of each patient was calculated. The mean dental age of the sample was 13.9 years (± 1.84) (Table 1).

**Table 1:** Mean chronological age and dental age in the study population (*n* = 194).

Chronological age	Mean	Std. deviation
	13.71	2.08
Dental age	13.93	1.84

### Correlation between chronological age and dental age

Data analysis revealed a significant positive correlation between chronological age and dental age, as assessed by the Demirjian method, among adolescents in Gachsaran. The overall Pearson correlation coefficient was 0.876 (*p* < 0.001). When analyzed by gender, the correlation coefficients were 0.894 for males and 0.876 for females (Table 2).

**Table 2:** Correlation between chronological age and dental age in the study population.

Gender				95% Confidence intervals (2-tailed)	
				Lower	Upper
Males	Chronological age * Dental age	Pearson correlation	0.894	0.815	0.941
		Sig. (2-tailed)	< 0.01		
		N	45		
Females	Chronological age * Dental age	Pearson correlation	0.876	0.833	0.909
		Sig. (2-tailed)	< 0.01		
		N	149		
Total	Chronological age * Dental age	Pearson correlation	0.876	0.838	0.905
		Sig. (2-tailed)	< 0.01		
		N	194		

**Fig 1:**
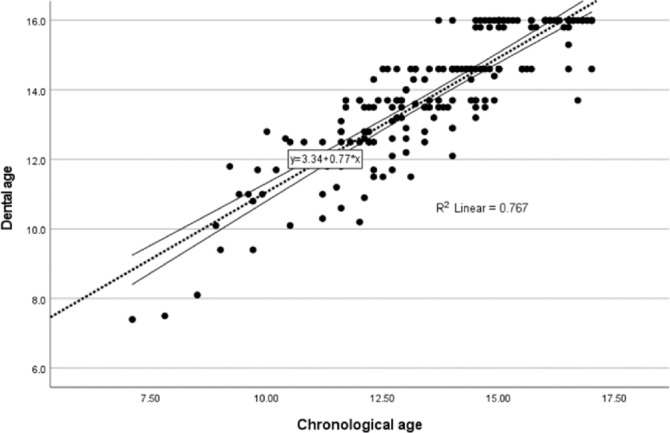
Scatter plot illustrating the relationship between chronological age and dental age, with a fitted linear regression line (R2=0.767)

During the analysis of the correlation between chronological age and dental age (using the Demirjian method), the following regression formula was derived (Fig. 1):

$$Dental{\text{ }}Age{\text{ }} = {\text{ }}0.77{\text{ }} \times {\text{ }}Chrono\log ical{\text{ }}Age{\text{ }} + {\text{ }}3.34$$

### Correlation between dental age and chronological age across CVMS stages

Among males, the highest correlation coefficient was 0.904, observed at CVMS1, indicating a strong positive correlation between chronological age and dental age. Correlation coefficients at other stages were lower, with some being weak or even negative (e.g., − 0.815 at CVMS6, which was not statistically significant) (Table 3).

Among females, the highest correlation coefficient was 0.887, also observed at CVMS1, reflecting a strong positive correlation similar to males. Correlation coefficients at other stages were lower, although several remained statistically significant (Table 3).

**Table 3:** Correlation between dental age and chronological age across different stages of cervical vertebral maturation, categorized by gender.

Gender	CVMS			
Males	1	Chronological age * Dental age	Pearson correlation	0.904
			Sig. (2-tailed)	0.035
			N	5
	2	Chronological age * Dental age	Pearson correlation	0.739
			Sig. (2-tailed)	0.261
			N	4
	3	Chronological age * Dental age	Pearson correlation	0.796
			Sig. (2-tailed)	0.000
			N	16
	4	Chronological age * Dental age	Pearson correlation	0.561
			Sig. (2-tailed)	0.148
			N	8
	5	Chronological age * Dental age	Pearson correlation	0.160
			Sig. (2-tailed)	0.705
			N	8
	6	Chronological age * Dental age	Pearson correlation	−0.815
			Sig. (2-tailed)	0.185
			N	4
Females	1	Chronological age * Dental age	Pearson correlation	0.887
			Sig. (2-tailed)	0.045
			N	5
	2	Chronological age * Dental age	Pearson correlation	0.812
			Sig. (2-tailed)	0.026
			N	7
	3	Chronological age * Dental age	Pearson correlation	0.579
			Sig. (2-tailed)	0.002
			N	25
	4	Chronological age * Dental age	Pearson correlation	0.618
			Sig. (2-tailed)	0.000
			N	29
	5	Chronological age * Dental age	Pearson correlation	0.745
			Sig. (2-tailed)	0.000
			N	51
	6	Chronological age * Dental age	Pearson correlation	0.730
			Sig. (2-tailed)	0.000
			N	32

### Correlation between dental age and skeletal age

Data analysis revealed a significant positive correlation between dental age and skeletal age in adolescents from Gachsaran. The overall Spearman correlation coefficient was 0.760 (*p* < 0.001). When analyzed by gender, the correlation coefficients were 0.779 for males and 0.785 for females (Table 4).

**Table 4:** Correlation between dental age and skeletal age (CVMS) in the study population.

Gender				95% Confidence intervals (2-tailed)	
				Lower	Upper
Male	Dental age * CVMS	Spearman’s$\:\rho\:$Correlation	0.779	0.624	0.875
		Sig. (2-tailed)	< 0.01		
		N	45		
Female	Dental age * CVMS	Spearman’s$\:\rho\:$Correlation	0.785	0.712	0.841
		Sig. (2-tailed)	< 0.01		
		N	149		
Total	Dental age * CVMS	Spearman’s$\:\rho\:$Correlation	0.760	0.691	0.815
		Sig. (2-tailed)	< 0.01		
		N	194		

### Correlation between chronological age and skeletal age

Data analysis revealed a significant positive correlation between chronological age and skeletal age among adolescents in Gachsaran. The overall Spearman correlation coefficient was 0.688 (*p* < 0.001). When analyzed by gender, the correlation coefficients were 0.783 for males and 0.707 for females (Table 5).

**Table 5:** Correlation between chronological age and skeletal age (CVMS) in the study population.

Gender				95% Confidence Intervals (2-tailed)	
				Lower	Upper
Males	Chronological age * CVMS	Spearman’s$\:\rho\:$correlation	0.783	0.631	0.878
		Sig. (2-tailed)	< 0.01		
		N	45		
Females	Chronological age * CVMS	Spearman’s$\:\rho\:$correlation	0.707	0.613	0.781
		Sig. (2-tailed)	< 0.01		
		N	149		
Total	Chronological age * CVMS	Spearman’s$\:\rho\:$correlation	0.688	0.603	0.757
		Sig. (2-tailed)	< 0.01		
		N	194		

### Average dental age and chronological age by CVMS stage

In males, the mean dental age was 13.8 years, while the mean chronological age was 13.75 years. Across all cervical vertebral maturation stages, the mean dental age was higher than the mean chronological age, except at stages I and II.

In females, the mean dental age was 13.97 years, compared to a mean chronological age of 13.7 years. In this group, greater consistency was observed, as the mean dental age exceeded the mean chronological age at all cervical vertebral maturation stages (Table 6).

**Table 6:** Mean dental age and chronological age at different stages of cervical vertebral maturation.

Gender	CVMS	N	Chronological age		Dental age	
			Mean	Std. deviation	Mean	Std. deviation
Males	1	5	10.82	1.86	10.56	1.58
	2	4	12.77	1.00	12.25	1.50
	3	16	12.99	1.82	13.16	1.81
	4	8	14.45	1.06	14.69	1.32
	5	8	15.89	0.72	15.95	0.09
	6	4	15.75	0.61	15.85	0.10
	Total	45	13.75	2.09	13.80	2.17
Females	1	5	9.08	1.19	9.78	1.67
	2	7	10.47	1.69	11.23	1.94
	3	25	12.11	1.30	12.56	1.05
	4	29	13.42	1.49	13.73	0.91
	5	51	14.42	1.40	14.63	1.02
	6	32	15.46	1.29	15.51	0.77
	Total	149	13.70	2.09	13.97	1.73

## Discussion

Selecting an orthodontic treatment plan, particularly for growth modification, requires understanding the patient’s growth potential, with chronological age often used as a reference. However, chronological age does not always reflect physical maturity due to factors such as premature birth, birth weight, and chronic illnesses^20,21^. This study investigated the relationship between chronological age and physical maturation, specifically focusing on the dental development and cervical vertebrae maturation across sexes, in order to provide optimal timing for orthodontic interventions despite the limitations of chronological age.

### Correlation between chronological age and dental age

In this study, the results show that the average dental age, as calculated by the Demirjian method, is 0.22 years (80 days) higher than the average chronological age in this population (Table 1). Recent studies have similarly examined the accuracy of dental age estimation methods, particularly the Demirjian method, and some have found that it tends to overestimate dental age^14,16^.,2317^17,23^, Similar to our study, Madalena et al^22^. (2023) reported that the Demirjian method overestimated dental age in a Brazilian pediatric population^22^. Similarly, Hedayati et al. (2014) and Bagherian et al. (2011) observed overestimation of dental age in Iranian pediatric populations^23,24^. In contrast, in an Indian pediatric population, Chowdhry et al.^25^(2023) demonstrated that the London Atlas method showed greater accuracy in dental age estimation^25^.

Our analysis revealed a strong positive Pearson correlation between dental age and chronological age. (*r* = 0.894 in males and *r* = 0.876 in females) (Table 2). Furthermore, Litsas et al. (2016) found high correlations between chronological and dental age in both sexes (*r* = 0.741 in males and *r* = 0.770 in females), which is in line with our findings^15^. in spite of this strong correlation, the mean advancement of 80 days should be considered when applying dental age for orthodontic treatment planning.

A linear regression analysis between chronological age and dental age (Demirjian method) demonstrated the formula: Dental Age = 0.77 × Chronological Age + 3.34 (R² = 0.767) (Fig. 1).This indicates a strong correlation between chronological and dental age in our sample, suggesting that chronological age can reasonably predict dental maturation.

### Correlation between dental age and chronological age across CVMS stages

Our findings indicate that the highest correlation between dental age and chronological age was observed at the first cervical vertebral maturation stage (CVMS1) for both males and females, reflecting a strong positive association (Table 3). This finding is clinically relevant, particularly for the management of Class III patients with a retruded maxilla, as treatment is recommended to be initiated during the CVMS1 or CVMS2 stages^12^. In agreement with our results, Rozylo-Kalinowska et al. (2011) also reported a significant correlation at CVMS1 in both sexes^26^. In contrast, Mollabashi et al. (2019) identified the highest correlation at CVMS5 in males and CVMS4 in females^27^. These discrepancies may be attributed to differences in sample size and population characteristics.

Also, in males a progressive decrease in the correlation between dental age and chronological age from CVMS1 to CVMS6 was observed, whereas in females the correlation decreased up to CVMS3 and subsequently increased. This sex-specific pattern may be attributed to differences in the timing and rate of skeletal maturation between sexes, with female generally reaching pubertal growth and skeletal maturity earlier than males^19,28^. Following the pubertal growth peak, dental maturation in females appears to show a more consistent relationship with chronological age, whereas in males, the prolonged and variable pattern of skeletal maturation may contribute to increasing discordance between dental and chronological age across advanced CVMS stages^17,28^. The smaller sample size of males in the present study should also be considered, as it may have reduced statistical power and affected the stability of correlation coefficients, particularly in the later stages of cervical vertebral maturation^29^.

Consistent with previous reports, sex-related differences were observed in the association between dental and chronological age across cervical vertebral maturation stages, reflecting differences in the progression of skeletal and dental development^12,30^.

### Correlation between dental age and skeletal age

In this study, a strong and statistically significant correlation was observed between dental age and skeletal age (ρ = 0.76), supporting the use of dental age as a useful indicator of skeletal maturation assessment (Table 4). In a Korean pediatric population, Ong et al. (2024) reported a relatively strong correlation between dental age and skeletal age (ρ = 0.826 in males and ρ = 0.883 in females), indicating a slightly stronger association compared with the present study conducted in Gachsaran^16^. Similarly, in a Romanian pediatric population, Ghergie M et al. (2025) demonstrated a strong and statistically significant correlation between cervical vertebral maturation and dental age assessed using the Demirjian method^30^. Likewise, Arbutina et al. (2025) reported a highly significant correlation between dental age and skeletal age^31^.

### Correlation between chronological age and skeletal age

A significant positive correlation was found between chronological age and skeletal maturity assessed by CVMS in the total sample (ρ = 0.688). The correlation was stronger in males (ρ = 0.783) than in females (ρ = 0.707), indicating a closer synchronization between chronological age and skeletal maturation in males (Table 5). Despite the significant associations, the moderate strength of correlation suggests that chronological age alone is insufficient for accurate assessment of skeletal maturity. Therefore, the use of CVMS as a complementary indicator is recommended for more reliable growth evaluation in clinical practice.

To achieve optimal outcomes in growth-modification treatments, accurate identification of cervical vertebral maturation stages II and III is crucial, as the pubertal growth spurt occurs during these stages. It is generally recommended to initiate treatment at stage II in males and before stage II in females^32^. Albu ŞD et al. (2024) further demonstrated that the mandibular growth spurt occurs in the year following CVMS III^33^.

Consistent with these findings, the present study precisely determined the mean chronological age for each stage of cervical vertebral maturation in each sex (Table 6). Our findings confirm that physical maturity occurs at different chronological age in each sex. The mean chronological age of males at CVMS2 and CVMS3 was 12.77 and 12.99 years, respectively, while for females, the corresponding ages were 10.47 and 12.11 years. These findings confirm that physical maturity occurs at different times in males and females, indicating that chronological age alone is not a reliable measure for planning growth-modifying treatments.

Overall, among the assessed parameters, dental age showed the strongest correlation with chronological age (*r* = 0.876), compared with the correlations between skeletal and chronological age (*r* = 0.688) and between dental and skeletal age (*r* = 0.760), suggesting that dental maturation may serve as a more consistent indicator of biological age in this population.

## Limitations & future directions

This study has some limitations. First, there was a gender imbalance, with more females (*n* = 149) than males (*n* = 45), which may affect generalizability. Second, all assessments were conducted by a single examiner; therefore, although intra-rater reliability was confirmed, inter-rater reliability could not be assessed. Third, some CVMS subgroups had small sample sizes, potentially limiting the power to detect subtle differences. Despite these limitations, the findings provide valuable insights into the relationship between chronological age and skeletal maturity.

AI-based approaches, particularly Convolutional Neural Networks (CNNs), offer a promising alternative to traditional methods like Demirjian’s, providing faster, automated, and potentially more accurate dental age estimation^34^. Future studies with larger and balanced cohorts are needed to validate these methods for clinical and forensic applications.

## Conclusion

This study demonstrated significant positive correlations between chronological age and both dental and skeletal maturation. Dental age assessed using the Demirjian method tended to overestimate chronological age in this population, underscoring the importance of population-specific interpretation. The strongest association between dental and chronological age was observed at CVMS1 in both sexes. Notably, dental age showed a stronger association with chronological age than skeletal maturity, suggesting that dental development may represent a more stable marker of biological age in this population. Overall, these findings indicate that chronological age alone is insufficient to accurately reflect biological maturity.

## Data Availability

The datasets used and/or analyzed during the current study available from the corresponding author on reasonable request.
